# Wnt signal transduction pathways: modules, development and evolution

**DOI:** 10.1186/s12918-016-0299-7

**Published:** 2016-08-01

**Authors:** Losiana Nayak, Nitai P. Bhattacharyya, Rajat K. De

**Affiliations:** 1 Indian Statistical Institute, 203 Barrackpore Trunk Road, Kolkata, 700108 India; 2 Saha Institute of Nuclear Physics, 1/AF Bidhan Nagar, Kolkata, 700064 India; 3 Present Affiliation: Biomedical Genomics Centre, 5 Suburban Hospital Road, Kolkata, 700020 India

**Keywords:** Module tree, Modularization algorithm, Wnt signal transduction pathway, Pathway phylogeny, Pathway development, Wnt gene family evolution

## Abstract

**Background:**

Wnt signal transduction pathway (Wnt STP) is a crucial intracellular pathway mainly due to its participation in important biological processes, functions, and diseases, i.e., embryonic development, stem-cell management, and human cancers among others. This is why Wnt STP is one of the highest researched signal transduction pathways. Study and analysis of its origin, expansion and gradual development to the present state as found in humans is one aspect of Wnt research. The pattern of development and evolution of the Wnt STP among various species is not clear till date. A phylogenetic tree created from Wnt STPs of multiple species may address this issue.

**Results:**

In this respect, we construct a phylogenetic tree from modules of Wnt STPs of diverse species. We term it as the ‘Module Tree’. A module is nothing but a self-sufficient minimally-dependent subset of the original Wnt STP. Authenticity of the module tree is tested by comparing it with the two reference trees.

**Conclusions:**

The module tree performs better than an alternative phylogenetic tree constructed from pathway topology of Wnt STPs. Moreover, an evolutionary emergence pattern of the Wnt gene family is created and the module tree is tallied with it to showcase the significant resemblances.

**Electronic supplementary material:**

The online version of this article (doi:10.1186/s12918-016-0299-7) contains supplementary material, which is available to authorized users.

## Background

Wnts are secreted lipid-modified glycoproteins rich with cysteine amino acid. They bind to Frizzled seven-transmembrane-spanning receptors (FZDs) along with co-receptor LRPs (Lipoprotein Receptor-related Proteins) and initiate a cascaded series of steps, which altogether are known as the Wnt STP [1, 2]. Wnt STP is involved in many crucial cellular functions, i.e., regulation of fate determination, proliferation, differentiation, migration and apoptosis of cells [3, 4]. In adult organisms, Wnts maintain stem cell like fates in the intestinal epithelium [5], skin [6], and hematopoietic cells [7], which entitles this pathway with immense possibility in regeneration and specification [8, 9], differentiation and wound healing [10, 11], and induced tissue creation [12] among others. A number of studies has been dedicated to analyze the specific details of Wnt STPs in a few model organisms [13–15]. On the contrary, only a few investigations have been initiated to understand how this pathway itself has developed and evolved [16, 17]. A phylogenetic tree constructed from modules may facilitate better understanding of Wnt STP development and evolution.

A module is a subset of a pathway/network, which is/tends to be self-sufficient and have minimal dependency on the rest part of the network. We divide a network into a number of modules, because complexity and size of each module is less than that of the entire pathway. So it becomes easy to study and understand the entire network by parts. A better operational view of a pathway can be had by analyzing its modules [18, 19]. Modules can also be compared among two species and their dissimilarity can be used as a measure of their distance. These distances among a set of species can be utilized to construct a phylogenetic tree namely the “Module Tree” [20].

Phylogenetic trees can also be generated from signal transduction pathway by their size [21, 22], similarity of the nucleotide sequences [23], amino acid sequences [24], enzyme sequences [25], and protein structural classification [26]. Some other ways of alignment are based on functional similarity of the enzymes [27] and proteins [28], enzyme hierarchy and gene ontology [29], chemical structures or compound similarity [30]. Presence of common topological structures, i.e., graphlets or sub-pathways [31], and presence or absence of pathways in an entire pathway repertoire [32] can also be utilized for pathway comparison and subsequently for phylogenetic tree construction.

Here, we have considered Wnt STPs of 48 species (Table 1) ranging from placozoans to humans to construct our module data based phylogenetic tree, i.e., module tree. As an extension, we have also considered Wnt STPs of 99 species (Table 4), later found in an updated KEGG/Pathway database [33]. Tables 1 and 4 list total number of genes present in the species-specific Wnt STPs, total number of modules created from a pathway, and the type and source of 18S rRNA sequence used [G: GenBank accession number (Complete sequence), G-P: GenBank accession number (Partial Sequence), G-S: GenBank accession number of predicted 18S rRNA sequence as given in SILVA database [34], and S-E: Sequence taken from Stage and EickBush, 2007 [35].

**Table 1:** A list of 48 species-specific Wnt signaling pathways and their respective 18S rRNA Reference ids

Sl.	KEGG	Binomial	Common	No. of	No. of	18S rRNA
No.	code	nomenclature	name	genes	modules	sequence id
1	aag	*A. aegypti*	Yellow fever	34	6	U65375 [G]
			mosquito			
2	aga	*A. gambiae*	Mosquito	31	6	AM157179 [G]
3	ame	*A. mellifera*	Honey bee	38	7	AY703484 [G-P]
4	aml	*A. melanoleuca*	Giant Panda	59	8	GL196163 [G]
5	api	*A. pisum*	Pea aphid	32	6	U27819 [G]
6	bfo	*B. floridae*	Florida lancelet	45	7	M97571 [G]
7	bmy	*B. malayi*	Filaria	34	7	AAQA01003643 [G-S]
8	bta	*B. taurus*	Cow	59	8	NR_036642 [G]
9	cbr	*C. briggsae*	-	23	3	FJ380929 [G]
10	cel	*C. elegans*	Nematode	23	3	EU196001 [G-P]
11	cfa	*C. familiaris*	Dog	58	8	AAEX02007663 [G-S]
12	cin	*C. intestinalis*	Sea squirt	42	7	AB013017 [G-P]
13	cqu	*C. quinquefasciatus*	Southern house	35	7	AAWU01013261 [G-S]
			mosquito			
14	dan	*D. ananassae*	-	37	7	XR_046314 [G]
15	der	*D. erecta*	-	37	7	XR_046906 [G]
16	dgr	*D. grimshawi*	-	37	7	[S-E]
17	dme	*D. melanogaster*	Fruit fly	37	7	M21017 [G]
18	dmo	*D. mojavensis*	-	37	7	XR_047783 [G]
19	dpe	*D. persimilis*	-	32	5	XR_046906 [G]
20	dpo	*D. pseudoobscura*	-	32	6	XR_053284 [G]
		*pseudoobscura*				
21	dre	*D. rerio*	Zebrafish	59	8	AC139725 [G-S]
22	dse	*D. sechellia*	-	37	7	XR_048770 [G]
23	dsi	*D. simulans*	-	22	5	AY037174 [G]
24	dvi	*D. virilis*	-	38	7	XR_049279 [G]
25	dwi	*D. willistoni*	-	37	7	XR_049811 [G]
26	dya	*D. yakuba*	-	36	6	XR_050457 [G]
27	ecb	*E. caballus*	Horse	56	8	AJ311673 [G-P]
28	gga	*G. gallus*	Chicken	54	8	M59389 [G]
29	hmg	*H. magnipapillata*	-	31	6	HQ392522 [G-P]
30	hsa	*H. sapiens*	Human	60	8	X03205 [G]
31	isc	*I. scapularis*	Black-legged	30	6	ABJB010180167 [G-S]
			tick			
32	mcc	*M. mulatta*	Rhesus Monkey	59	8	FJ436026 [G-P]
33	mdo	*M. domestica*	Opossum	55	7	AJ311676 [G-P]
34	mmu	*M. musculus*	Mouse	60	8	X00686 [G]
35	nve	*N. vectensis*	Sea anemone	33	6	AF254382 [G]
36	nvi	*N. vitripennis*	Jewel wasp	38	7	GQ410677 [G-P]
37	oaa	*O. anatinus*	Platypus	47	7	AJ311679 [G-P]
38	phu	*P. humanus*	Human body	37	7	FJ267399 [G-P]
		*corporis*	louse			
39	ptr	*P. troglodytes*	Chimpanzee	58	8	AADA01268803 [G-S]
40	rno	*R. norvegicus*	Rat	60	8	X01117 [G]
41	smm	*S. mansoni*	-	25	5	U65657 [G]
42	spu	*S. purpuratus*	Purple	41	6	L28055 [G]
			sea urchin			
43	ssc	*S. scrofa*	Pig	21	4	AY265350 [G]
44	tad	*T. adhaerens*	-	23	6	Z22783 [G]
45	tca	*T. castaneum*	Red flour	37	7	HM156711 [G-P]
			beetle			
46	tgu	*T. guttata*	Zebra finch	47	7	ABQF01063677 [G-S]
47	xla	*X. laevis*	African	51	8	X04025 [G]
			clawed frog			
48	xtr	*X. tropicalis*	Western	57	8	AAMC01103672 [G-S]
			clawed frog			

Notations: G: GenBank accession number (Complete sequence), G-P: GenBank accession number (Partial Sequence), G-S: GenBank accession number of predicted 18S rRNA sequence as given in SILVA database (Pruesse et al. 2007), S-E: Sequence taken from (Stage and Eickbush 2007)

In this article we have created two alternative phylogenetic trees, i.e., the module tree and the pathway tree, to study development of Wnt STPs. These trees have been created by considering modules and whole pathway topology of species specific Wnt STPs respectively. Four species sets corresponding to 99, 48, 29 and 12 species have been considered. These phylogenetic trees represent development of the Wnt STP obtained at module and pathway level. They were compared with the NCBI taxonomy and 18S rRNA trees for their quality assessment in representing development of Wnt STP. The phylogenetic trees have been created with MEGA version 4.0.2 [36]. In addition, an evolutionary emergence pattern of the Wnt gene family has been constructed and the module tree from 48 species-specific Wnt STPs has been tallied with it to showcase the resemblances. A large diverse species set (99 species) has been avoided as it involves a set of different extensive phylum specific studies which is beyond the scope of this manuscript.

## Data

Species-specific Wnt STPs in KEGG/Pathway database [33] has been taken as raw data (Table 1). The pathway specific interactions were extracted from their corresponding KGML (KEGG Markup Language) files. The database uses a unique three (four in some cases) letter code for each species along with their biological and common names (wherever applicable), i.e., ‘hsa’ for *H. sapiens* (human). These three/four letter codes have been used extensively in this manuscript.

### 18S rRNA Sequence Data

18S rRNA is a component of small eukaryotic ribosomal subunit (40S). 18S rRNA sequences have slow evolutionary rate. Hence, they are widely used in reconstructing the evolutionary history and ancient divergences of organisms. Here, most of the 18S rRNA sequences have been taken from GenBank [37] for construction of the 18S rRNA tree. With a simple search dialogue of “— [organism] AND 18S ribosomal RNA [keyword] NOT (partial)”, the sequence of interest can be extracted easily. If complete sequences are not available, the “NOT (partial)” dialogue can be omitted and a search for partial sequences can be done.

We have found 28 complete and 11 partial 18S rRNA nucleotide sequences for which GenBank accession numbers are listed in Table 1. Eight sequences have been taken from the SILVA comprehensive ribosomal RNA databases (http://www.arb-silva.de/). SILVA [34] provides quality checked and aligned free ribosomal RNA sequence data for academic use. It has datasets of aligned small (16S/18S, SSU) and large subunit (23S/28S, LSU) rRNA sequences for Bacteria, Archaea and Eukarya. We have taken sequences from the SSU r106 database and their respective GenBank accession numbers are given in Table 1. 18S rRNA sequence of *D. grimshawi* has been taken from Stage and Eickbush, 2007 [35] as it is not available in GenBank or SILVA.

## Methods

**Fig. 1:**
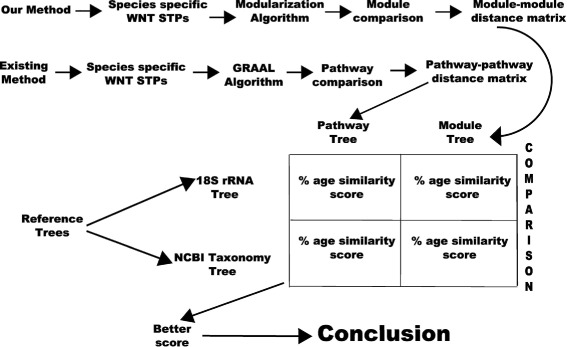
Pipeline of the methodology

Here we describe the methodology (Fig. 1) involved in creating phylogenetic trees from species specific Wnt STPs, taxonomy information from NCBI and 18S rRNA sequences. We have considered two different sets of factors to do system level development analysis of species-specific Wnt STPs. Our aim is to know the similarity in percentage of our constructed phylogenetic trees (constructed from pathway topology and modules) with respect to the standard evolutionary trees (18S rRNA and NCBI taxonomy tree).

### Module tree method

The module tree has been generated solely based on one-to-one mapping of members present in modules of different species-specific Wnt STPs. A module can be defined as a subset of a STP that tends to be self-sufficient by maintaining minimal dependency on the remaining part of the STP. Modules have been created by the Modularization Algorithm developed earlier by the authors [18] based on a user defined factor *c*. The lowest possible *c*-value is always 1. The upper limit of *c*-value is the highest total degree of a node present in the considered network. Detailed working procedure of the algorithm can be obtained by following the pseudo-code furnished here.



Number of modules found in a pathway depends on its size and complexity. Hence, varying number of modules can be found for different species-specific Wnt signal transduction pathways. Presence, absence or modification (increase/decrease due to addition/deletion of nodes) found in modules of a pair of species-specific pathways represent their distance. This approach is inspired by the NCE method described by Heymans and Singh, 2003 [27]. NCE method detects number of common enzymes between two pathways and tries to guess similarity based on that number. Here, rather than considering number of common enzymes, we are calculating number of common nodes present in the corresponding module of two species-specific Wnt signal transduction pathways. The score is then normalized by dividing it with the total number of non-redundant nodes present in both the species-specific modules. Hence, if pathway of species *x* has *m*_1_ modules and pathway of species *y* has *m*_2_ modules, we will get a similarity matrix of order *m*_1_×*m*_2_. Each element of the matrix represents similarity score between two different modules belonging to two different species (Eq. 1). Let *M*_1_ be a module of species *x*, i.e., *M*_1_ is the set of all the nodes in the module. Similarly, in a species *y*, *M*_2_ is a set of nodes that constitute a module. The score of similarity *Sim*(*M*_1_,*M*_2_) between module *M*_1_ of species *x* and module *M*_2_ of species *y* is defined as

1 $$ Sim(M_{1},M_{2}) = |M_{1} \cap M_{2}| / |M_{1} \cup M_{2}|  $$

Now the similarity *S*_*M*_(*x,y*) between species *x* and *y* is defined as

2 $$ S_{M}(x,y) = \sum^{m_{1},m_{2}}_{i=1,j=1}Sim(M_{i},M_{j})/(m_{1} \times m_{2})  $$

Distance score *D*_*M*_(*x,y*) is defined as

3 $$ D_{M}(x,y) = 1 - S_{M}(x,y)  $$

These distance scores found among 48 different species are utilized for creation of the module tree. Our purpose in creating such a tree is to test its novelty in presenting the pathway’s development.

### Pathway tree method

We have generated the pathway tree based on topological distances among species-specific pathways. Topological distance *D*_*P*_(*x,y*) between two pathways of species *x* and *y* has been defined as *D*_*P*_(*x,y*)=1−*S*_*P*_(*x,y*) where *S*_*P*_(*x,y*) has been the topological similarity between the two species-specific pathways. *S*_*P*_(*x,y*) has been calculated by the GRAph ALigner algorithm (GRAAL) developed by Kuchaiev et al. [21] and implemented in the GraphCrunch2 software [38]. *S*_*P*_(*x,y*) is nothing but Edge Correctness (EC) value between a pair of species-specific Wnt STPs. Edge correctness is the percentage of edges in the first graph that are aligned to edges in the second graph. High edge correctness means the pair of networks considered share similar topologies.

GRAAL [21] performs network alignment by using topological information based on graphlets of individual networks. Given two networks, the GRAAL algorithm (a seed-and-extend algorithm) finds an embedding of the smaller network into the larger network. It greedily aligns nodes based on their signature similarities while traversing both networks simultaneously in a breadth-first manner. Every node in the smaller network gets aligned to exactly one node in the larger one and finally a topological similarity score gets generated.

### 18s rRNA tree method

**Fig. 2:**
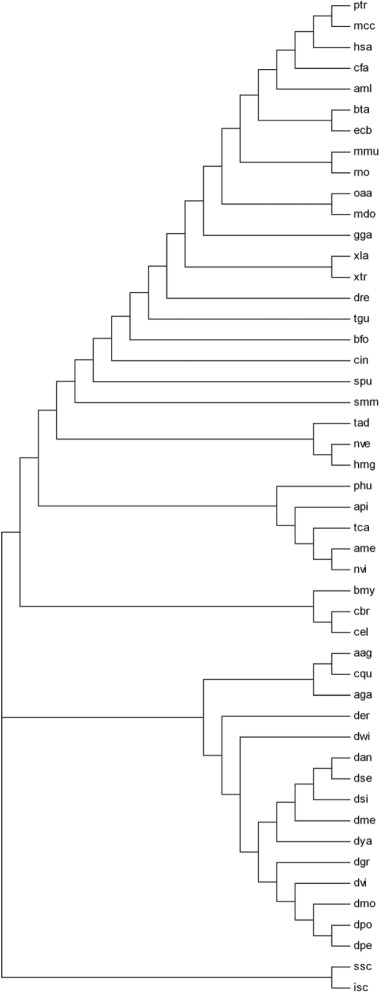
The 18S rRNA tree

Standard 18S rRNA sequences (Table 1) have been used to create the 18S rRNA tree as shown in Fig. 2. The evolutionary history has been inferred using the Neighbor-Joining method [39]. The optimal tree with the sum of branch length = 2.18407422 has been considered. The evolutionary distances have been computed in the units of the number of base substitutions per site using the Maximum Composite Likelihood method [40]. All positions containing gaps and missing data have been eliminated only in pairwise sequence comparisons (Pairwise deletion option). A total of 2687 positions were there in the final dataset.

### NCBI taxonomy tree method

**Fig. 3:**
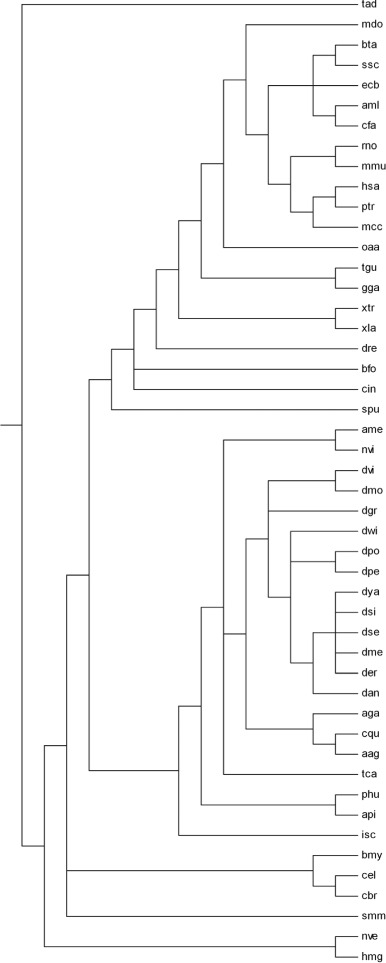
The NCBI taxonomy tree

The NCBI taxonomy tree (Fig. 3) has been created with the help of NCBI taxonomy database (http://www.ncbi.nlm.nih.gov/Taxonomy/CommonTree/wwwcmt.cgi) [41]. Newick format of the tree has been saved as a text tree after adding organism names in the “Taxonomy Common tree” page.

### Tree comparison method

Nye et al. [42] have developed an algorithm that pairs up each branch in one phylogenetic tree with a matching branch in the second one, and finds the optimum one-to-one map between branches in the two trees in terms of a topological score. They have developed an Java applet (http://www.mas.ncl.ac.uk/~ntmwn/phylo_comparison/pairwise.html) which enables one to explore the corresponding mapping between the phylogenetic trees interactively, and clearly highlights similar/different parts of the trees, both in terms of topology and branch length. Here, we have considered topology mainly.

Let us now describe the algorithm that compares two phylogenetic trees created from the same set of species. Given two phylogenetic trees *T*_1_ and *T*_2_ that share the same set of leaves *L*, the algorithm firstly assigns a score *s*(*i,j*) to every pair of edges (*i,j*) with *i*∈*T*_1_ and *j*∈*T*_2_. Then it pairs up branches in the two trees to optimize the overall score. This is equivalent to finding a bijection (i.e., a one-to-one and onto correspondence) *f*:*T*_1_→*T*_2_ between the branches of the trees that maximizes the quantity $\sum _{i \in T_{1}}S(i,f(i))$.

## Results and discussions

**Fig. 4:**
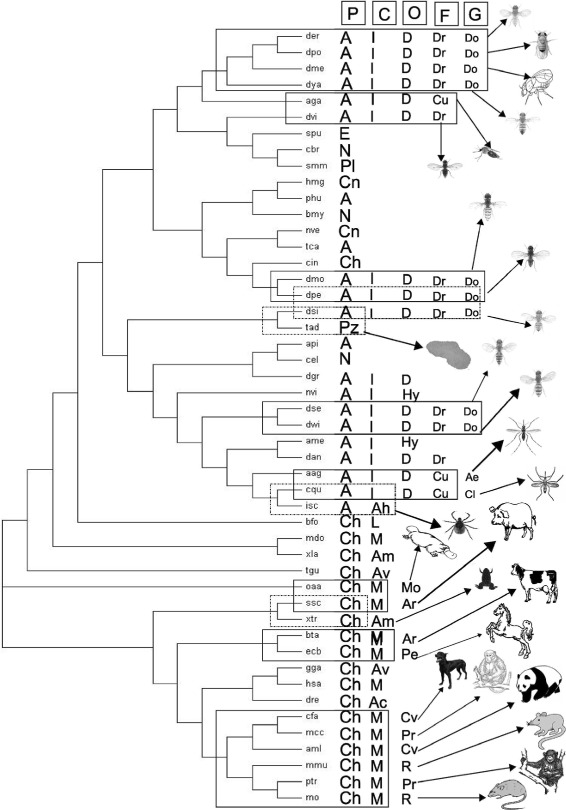
The module tree. It is constructed from 48 species. The notations used in the figure are listed in Table 2

**Fig. 5:**
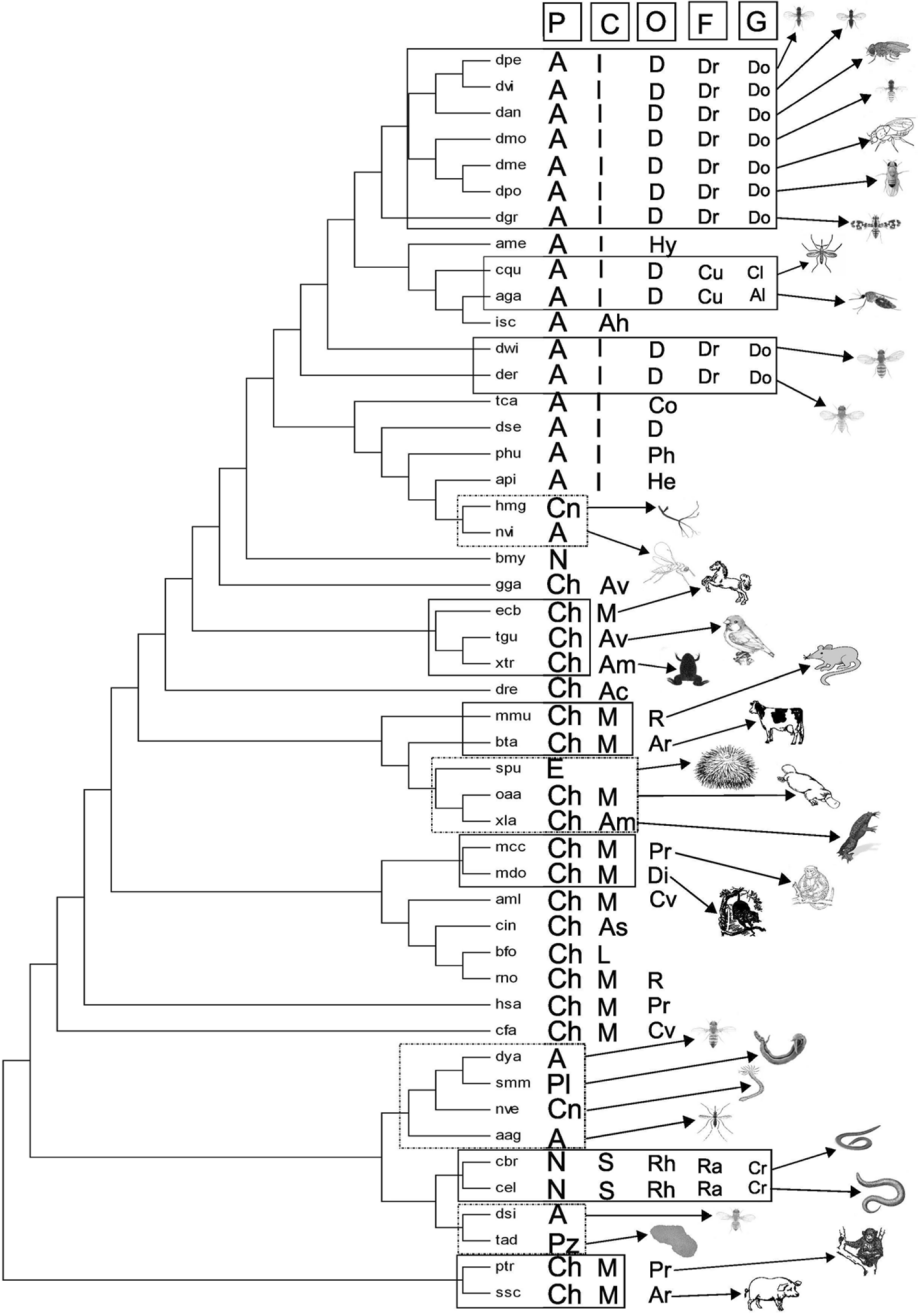
The pathway tree. It is constructed from 48 species. The notations used in the figure are listed in Table 2

In this section, we describe ranks and positional significance of the species in the pathway tree (Fig. 4) and the module tree (Fig. 5). The species with similar taxonomic ranks coming under the same clade have been marked by continuous rectangles. The species coming under different clades despite having similar taxonomy have been marked by dotted rectangles. While discussing positional significance of species, we have furnished the similarities in terms of taxonomic ranks (phylum, class and others) to the lowest possible taxonomic rank. Throughout this manuscript we have used some common notations while analyzing the phylogenetic trees. The notations are listed alphabetically in Table 2.

**Table 2:** List of notations used in Figs. 4 and 5

Notation	Rank	Name	Notation	Rank	Name
P	Phylum	-	R	Order	Rodentia
A	Phylum	Arthropoda	Ar	Order	Artiodactyla
Cn	Phylum	Cnidaria	Pe	Order	Perissodactyla
N	Phylum	Nematoda	Pr	Order	Primates
Ch	Phylum	Chordata	Aa	Order	Anura
E	Phylum	Echinodermata	Di	Order	Didelmorphia
Pl	Phylum	Platyhelminthes	Cv	Order	Carnivora
Pz	Phylum	Placozoa	Rh	Order	Rhabditida
C	Class	-	F	Family	-
I	Class	Insecta	Dr	Family	Drosophilidae
Ah	Class	Arachnida	Mu	Family	Muridae
Av	Class	Aves	Pi	Family	Pipidae
Am	Class	Amphibia	Cd	Family	Canidae
Ac	Class	Actinopterygii	U	Family	Ursidae
M	Class	Mammalia	Cu	Family	Culicidae
As	Class	Ascidiacea	Ra	Family	Rhabditidae
L	Class	Leptocardii	G	Genus	-
S	Class	Secernentea	Do	Genus	Drosophila
O	Order	-	Ms	Genus	Mus
D	Order	Diptera	Rt	Genus	Rattus
Hy	Order	Hymenoptera	X	Genus	Xenopus
Mo	Order	Monotremata	Ae	Genus	Aedes
Co	Order	Coleoptera	Cl	Genus	Culex
Ph	Order	Phthiraptera	Al	Genus	Anophelinae
He	Order	Hemiptera	Cr	Genus	Caenorhabditis

We have compared Wnt STPs of 48 different species as provided in Table 1 for creating a module tree (Fig. 4) and a pathway tree (Fig. 5). The considered species belong to seven different phyla, most of which (21) belong to the phylum Arthropoda followed by Chordata (19), Nematoda (3), Cnidaria (2), and single species from phyla Echinodermata, Placozoa and Platyhelminthes. As expected, some species have been placed closely in the tree following their taxonomic ranks. On the other hand, we have also found some deviations.

### Finding a better tree

Closely related species come under a single clade in both the module and pathway trees. In this sense, they have shown preservation of contemporary notions regarding development of species. But, they have shown numerous deviations also, when evolutionarily distant species came under a single clade or species having similar taxonomy got included in different clades. In order to put a universal measure to their quality, and to determine the better tree that represents development of Wnt STP, we have followed the concept of alternative phylogenetic tree comparison [42]. A brief description of this method of comparison is furnished in the methodology section.

We have compared the module and pathway trees with the reference trees (NCBI taxonomy tree and 18S rRNA tree). The reference trees represent phylogeny of living organisms from multiple point-of-views while the phylogenetic trees derived from species-specific Wnt STPs solely represent development of Wnt STP over the taken set of species. So a huge gap can be noticed among the two sets of phylogenetic trees in terms of similarity percentage. Still, a more similar tree is better than a less similar tree for development analysis.

The module tree has showed 42.4 % topological similarity with the NCBI taxonomy tree and 42.2 % similarity with the 18S rRNA tree followed by the pathway tree (38.2 % and 37.1 % respectively) as given in Table 3. Hence, the module tree has outperformed the pathway tree in representing Wnt STP development. However, among 48 18S rRNA sequences, 11 are partial and 8 are predicted. Did the incomplete sequences influence the 18S rRNA tree (e.g. two unrelated species are clustered together because of lack of the same part of 18s rRNA sequences)? To avoid such a notion, we have repeated our protocol with a species set of 29 species (aag, aga, aml, api, bfo, bta, cbr, dan, der, dgr, dme, dmo, dpe, dpo, dse, dsi, dvi, dwi, dya, gga, hsa, mmu, nve, rno, smm, spu, ssc, tad and xla), for which complete 18S rRNA sequences are available as given in Table 1. The module tree has also outperformed the pathway tree, for these species.

**Table 3:** Similarities among trees for 99, 48, 29 & 12 species in percentage

	99 species		48 species		29 species		12 species	
	NCBI	18S	NCBI	18S	NCBI	18S	NCBI	18S
	Taxonomy tree	rRNA tree	Taxonomy tree	rRNA tree	Taxonomy tree	rRNA tree	Taxonomy tree	rRNA tree
Module tree	36.3	32.4	42.4	42.2	48.9	39	45.4	55.4
Pathway tree	30.3	26.9	38.2	37.1	38.2	36.2	36.7	37.3

Some of the pathways in our species set comprising 48 species-specific pathways are partially known as the underlying graph structures of pathways (KEGG) are highly incomplete. Some model organisms are better studied than others. This could cause a bias and the pathway-based graph may be more prone to this bias. To avoid such a bias, and in order to strengthen our results, we have considered Wnt STP of a smaller and more complete pathway species set of 12 (aml, bta, cfa, dre, ecb, hsa, mcc, mdo, mmu, ptr, rno and xtr). These pathways have varying number (55–60) of nodes. For this species set too, the module tree has showed maximum similarity with the NCBI taxonomy tree (45.4 %) and the 18S rRNA tree (55.4 %) as given in Table 3.

The recently updated KEGG/Pathway database [33] lists 108 species-specific Wnt signaling pathways, out of which we have taken 99 pathways based on the availability of their respective 18S rRNA sequences. For the remaining 9 species (*P. tigris altaica*, *C. ferus*, *G. fortis*, *F. albicollis*, *P. humilis*, *C. cornix*, *F. peregrinus*, *C. mydas* and *P. bivittatus*), we could not find their respective 18S rRNA sequences. The list and details of the 99 considered species are given in Table 4. The module tree has out-performed the pathway tree for this diverse 99 species set. It has 36.3 % similarity with the NCBI taxonomy tree while the pathway tree has 30.3 % similarity. Moreover, the module tree has 32.4 % similarity with the 18S rRNA tree while the pathway tree has 26.9 % similarity as given in Table 3. The NCBI taxonomy, 18S rRNA, pathway and module trees for 99 species are provided in Table 4. The associated four figures for NCBI taxonomy, 18S rRNA, pathway and module trees and their respective newick formats are given in Additional file 1. The higher similarity of the module tree for all the species sets with the NCBI taxonomy and 18S rRNA trees suggests that the module tree is a better tree for studying Wnt signaling pathway development.

However evolution of Wnt gene family is a different phenomenon and the following subsections have concentrated on finding some links between the module tree for 48 species-specific pathways and the Wnt gene family evolutionary trend. The module tree of a reasonable size (48 species) has been taken to facilitate ease of linking it with the evolutionary trend. A large diverse species set (99 species) has been avoided as it involves a set of different extensive phylum specific studies which is beyond the scope of this manuscript.

**Table 4:** A list of 99 species-specific Wnt signaling pathways and their respective 18S rRNA Reference ids

Sl.	KEGG	Binomial	Common	No. of	No. of	18S rRNA
No.	code	nomenclature	name	genes	modules	sequence id
1	aag	*A. aegypti*	Yellow fever	41	6	U65375.1 [G]
			mosquito			
2	acs	*A. carolinensis*	Green anole	61	9	AY859624.1 [G-P]
3	aec	*A. echinatior*	Panamanian	36	6	AEVX01007365 [G-S]
			leafcutter ant			
4	aga	*A. gambiae*	Mosquito	39	6	AM157179.1 [G]
5	ame	*A. mellifera*	Honey bee	43	7	AB126807.1 [G-P]
6	amj	*A. mississippiensis*	American	62	9	AF173605.1 [G]
			alligator			
7	aml	*A. melanoleuca*	Giant Panda	62	9	ACTA01092993 [G-S]
8	api	*A. pisum*	Pea aphid	42	8	U27819.1 [G]
9	apla	*A. platyrhynchos*	Mallard	56	8	AF173614.1 [G]
10	aqu	*A. queenslandica*	Sponge	32	6	EF654521.1 [G-P]
11	asn	*A. sinensis*	Chinese	59	9	JX481969.1 [G-P]
			alligator			
12	bacu	*B. acutorostrata*	Mink whale	63	9	ATDI01000034 [G-S]
		*scammoni*				
13	bfo	*B. floridae*	Florida	42	7	M97571.1 [G]
			lancelet			
14	bmor	*B. mori*	Domestic	46	8	DQ347470.1 [G]
			silkworm			
15	bmy	*B. malayi*	Filaria	38	7	AF036588.1 [G-P]
16	bom	*B. mutus*	Wild yak	63	9	AGSK01136783 [G-S]
17	bta	*B. taurus*	Cow	63	9	NR_036642.1 [G]
18	cbr	*C. briggsae*	-	31	5	U13929.1 [G-P]
19	cel	*C. elegans*	Nematode	27	4	NR_000054.1 [G]
20	cfa	*C. familiaris*	Dog	63	9	AY623831.1 [G-P]
21	cfo	*C. floridanus*	Florida	42	7	AEAB01019515 [G-S]
			carpenter ant			
22	cge	*C. griseus*	Chinese hamster	63	9	NR_045132.1 [G]
23	chx	*C. hircus*	Goat	61	9	DQ149973.1 [G-P]
24	cin	*C. intestinalis*	Sea squirt	42	7	AB013017.1 [G-P]
25	cjc	*C. jacchus*	White tufted	62	9	AB571241.1 [G-P]
			ear marmoset			
26	clv	*C. livia*	Rock pigeon	56	8	AF173630.1 [G]
27	cmk	*C. milii*	Elephant shark	58	8	AY049813.1 [G]
28	cqu	*C. quinquefasciatus*	Southern house	41	6	AAWU01003351 [G-S]
			mosquito			
29	crg	*C. gigas*	Pacific oyster	45	8	AB064942.1 [G]
30	dan	*D. ananassae*	-	42	7	XR_046314.1 [G]
31	der	*D. erecta*	-	43	7	XR_046906.1 [G]
32	dgr	*D. grimshawi*	-	43	7	[S-E]
33	dme	*D. melanogaster*	Fruit fly	42	7	NR_133559.1 [G]
34	dmo	*D. mojavensis*	-	42	6	XR_047783.1 [G]
35	dpe	*D. persimilis*	-	39	6	XR_048244.1 [G]
36	dpo	*D. pseudoobscura*	-	41	7	XR_053284.1 [G]
		*pseudoobscura*				
37	dre	*D. rerio*	Zebrafish	63	9	AC139725 [G-S]
38	dse	*D. sechellia*	-	42	7	XR_048770.1 [G]
39	dsi	*D. simulans*	-	34	6	AY037174.1 [G]
40	dvi	*D. virilis*	-	43	7	XR_049279.1 [G]
41	dwi	*D. willistoni*	-	43	7	XR_049811.1 [G]
42	dya	*D. yakuba*	-	43	7	AAEU02010701 [G-S]
43	ecb	*E. caballus*	Horse	62	9	NR_046271.1 [G]
44	fca	*F. catus*	Domectis cat	62	9	AY150542.1 [G-P]
45	fch	*F. cherrug*	Saker falcon	56	8	AKMU01028905 [G-S]
46	gga	*G. gallus*	Chicken	57	9	AF173612.1 [G]
47	ggo	*G. gorilla gorilla*	Western lowland	62	9	CABD030100652 [G-S]
			gorilla			
48	hgl	*H. glaber*	Naked mole rat	63	9	AHKG01114378 [G-S]
49	hmg	*H. magnipapillata*	-	30	6	ABRM01041397 [G-S]
50	hro	*H. robusta*	-	42	8	AMQM01008875 [G-S]
51	hsa	*H. sapiens*	Human	63	9	X03205.1 [G]
52	hst	*H. saltator*	Jerdon’s	42	7	AEAC01025389 [G-S]
			jumping ant			
53	isc	*I. scapularis*	Black-legged	44	8	ABJB010537244 [G-S]
			tick			
54	lcm	*L. chalumnae*	Coelacanth	61	9	L11288.1 [G]
55	lgi	*L. gigantea*	Owl limpet	34	7	FJ977632.1 [G-P]
56	loa	*L. loa*	Eye worm	41	8	DQ094173.1 [G-P]
57	lve	*L. vexillifer*	Yangtze River	63	9	AUPI01105851 [G-S]
			dolphin			
58	mcc	*M. mulatta*	Rhesus Monkey	62	9	FJ436026 [G-P]
59	mcf	*M. fascicularis*	Crab eating macaque	63	9	AB172927 [G-S]
60	mde	*M. domestica*	House fly	42	7	GQ465780.1 [G-P]
61	mdo	*M. domestica*	Opossum	62	9	AJ311676.1 [G-P]
62	mgp	*M. gallopavo*	Turkey	56	8	AJ419877.1 [G]
63	mmu	*M. musculus*	Mouse	63	9	NR_003278.3 [G]
64	myb	*M. brandtii*	Brandt’s bat	61	9	ANKR01250841 [G-S]
65	myd	*M. davidii*	-	61	9	ALWT01111512 [G-S]
66	mze	*M. zebra*	Zebra mbuna	61	9	GBAN01001852 [G-S]
67	ngi	*N. galili*	Upper Galilee	63	9	JO020273 [G-S]
			mountains blind			
			mole rat			
68	nle	*N. leucogenys*	Northern white	63	9	ADFV01131837 [G-S]
			cheeked gibbon			
69	nve	*N. vectensis*	Sea anemone	30	6	AF254382.1 [G]
70	nvi	*N. vitripennis*	Jewel wasp	42	7	GQ410677.1 [G-P]
71	oaa	*O. anatinus*	Platypus	54	8	AJ311679.1 [G-P]
72	oas	*O. aries*	Sheep	63	9	AY753190.1 [G-P]
73	ocu	*O. cuniculus*	Rabbit	63	9	NR_033238.1 [G]
74	ola	*O. latipes*	Japanese medaka	59	9	AB105163.1 [G]
75	pale	*P. alecto*	Black flying fox	63	9	ALWS01159237 [G-S]
76	phd	*P. hodgsonii*	Chiru	61	9	AGTT01252085 [G-S]
77	phu	*P. humanus*	Human body	41	7	AF139482.1 [G-P]
		*corporis*	louse			
78	pon	*P. abelii*	Sumatran	63	9	ABGA01173767 [G-S]
			orangutan			
79	pps	*P. paniscus*	Bonobo	63	9	AJFE01002621 [G-S]
80	pss	*P. sinensis*	Chinese soft	60	9	JX481969.1 [G-P]
			shelled turtle			
81	ptr	*P. troglodytes*	Chimpanzee	63	9	AADA01153094 [G-S]
82	pxy	*P. xylostella*	Diamondback moth	32	5	JX390653.1 [G-P]
83	rno	*R. norvegicus*	Rat	63	9	NR_046237.1 [G]
84	rro	*R. roxellana*	Golden snub	63	9	JABR01093782 [G-S]
			nosed monkey			
85	shr	*S. harrisii*	Tasmanian devil	61	9	AFEY01231219 [G-S]
86	smm	*S. mansoni*	-	26	4	M62652.1 [G]
87	soc	*S. invicta*	Red fire ant	43	7	AY334566.1 [G-P]
88	spu	*S. purpuratus*	Purple	46	6	L28056.1 [G]
			sea urchin			
89	ssc	*S. scrofa*	Pig	62	9	AY265350.1 [G]
90	tad	*T. adhaerens*	-	26	5	ABGP01001110 [G-S]
91	tca	*T. castaneum*	Red flour	43	7	HM156711.1 [G-P]
			beetle			
92	tgu	*T. guttata*	Zebra finch	56	8	ABQF01063677 [G-S]
93	tru	*T. rubripes*	Torafugu	60	9	AB437886.1 [G-P]
94	tsp	*T. spiralis*	-	39	8	AY497012.1 [G-P]
95	tup	*T. chinensis*	Chinese tree shrew	62	9	ALAR01203917 [G-S]
96	umr	*U. maritimus*	Polar bear	60	9	AVOR01047284 [G-S]
97	xla	*X. laevis*	African	54	9	X04025.1 [G]
			clawed frog			
98	xma	*X. maculatus*	Southern platyfish	59	9	KJ774770.1 [G-P]
99	xtr	*X. tropicalis*	Western	60	9	AAMC02038921 [G-S]
			clawed frog			

Notations: G: GenBank accession number (Complete sequence), G-P: GenBank accession number (Partial Sequence), G-S: GenBank accession number of predicted 18S rRNA sequence as given in SILVA database (Pruesse et al. 2007), S-E: Sequence taken from (Stage and Eickbush 2007)

### Evolution of Wnts

The origin of Wnts and emergence of multicellularity are associated events. The comb jellies, sponges and placozoans of prebilaterian lineage possess a few Wnt genes [43]. But the Most Recent Common Ancestor (MRCA) of Cnidaria and Bilateria is believed to have an enormous expansion in ligand diversity [44], resulting in the origin of 11 of the 13 contemporary subfamilies. Wnt genes constitute a large family of lipid-modified, secreted signaling molecules. they are found to be highly conserved across the metazoan kingdom. Orthologs of individual Wnts have been found in animal species ranging from Cnidaria and Porifera (sponges) to flies and vertebrates. Their evolution spans approximately 600 million years of time [45].

The Wnt signal transduction pathway (STP) regulates various processes of metazoan development [44] and shows evolutionary conservation across a wide range of Metazoans [45, 46]. Wnt STP is a combination of three pathways, i.e., canonical Wnt, planar cell polarity (PCP) and Wnt/Ca ^2+^ STPs. The canonical Wnt STP is more conserved across metazoan species than the others [46]. The PCP Wnt STP (first discovered in fruitfly) controls epithelial planar polarity within the eye, wing, and thorax [47, 48]. It is well-conserved at molecular and functional levels throughout the bilaterian lineage [46]. The Wnt/Ca ^2+^ STP has been discovered in Xenopus and zebrafish [49, 50]. But no equivalent pathway has been found in any other model system possibly making it a vertebrate-specific pathway. Emergence of metazoans appear to be linked with all the three STPs. These STPs have been found well-conserved among a lot of metazoans, from cnidarians to vertebrates [46], based on the identification of their individual components.

#### Placozoa

They are the simplest in structure of all non-parasitic multicellular animals (Metazoa). They are generally classified as a single species, *Trichoplax adhaerens*. Components of a complete Wnt/ *β*-catenin STP associated with axial patterning of demosponge larvae [51], bilaterians and cnidarians [52] are present in Trichoplax [53].

#### Cnidaria

Cnidarians are the first metazoans with a defined adult body plan, main body axis, tissue level of organization and nervous system. They provide potential information regarding evolution of Wnt STP and its role in axis formation, polarity and germ-layer specification among others. Components of all the three Wnt STPs are present in cnidarians. They possess 14 Wnt orthologs [54, 55] of twelve distinct subfamilies including WntA whose human counterpart is absent. Multiple Wnt and Fzd genes have been found in *Nematostella vectensis*. So, expansion of these gene families must have occurred in early evolution. Out of total 13 Wnt sub-families, 12 have been retained in this species [56], while Wnt9 gene is not found. The large inventory of Wnt genes in Nematostella indicates diversification of this gene family in the cnidarian–bilaterian ancestor [44].

#### Platyhelminthes

Flatworms have a highly reduced and dispersed complement of Wnt STP. It includes orthologs of Wnt1, Wnt2, Wnt4, Wnt5 and Wnt11 subfamilies and a few paralogs in parasitic flatworms (5/6 genes). All the antagonists, receptors and key binding domains of Wnt STP are intact in this phylum indicating functional status of the canonical (Wnt/ *β*-catenin) and non-canonical (PCP and Wnt/Ca ^2+^) STPs. Evolution of flatworms appears to be associated with loss of Wnt6, Wnt7, Wnt8, Wnt9, Wnt10, Wnt16 and WntA subfamilies. Moreover, loss of Wnt4 paralogs is associated with the evolution of parasitism in this phylum [57].

It was found through in silico approaches that the *Hymenolepis microstoma* genome contains a total of six Wnt genes, representing five subfamilies, i,e., Hm-Wnt1, Hm-Wnt2, Hm-Wnt4, Hm-Wnt5 and Hm-Wnt11a/b]. Other down-stream members are also present, i.e., Hm-fzdB and Hm-dsh; Hm-Bcat; Hm-TCF/LEF. In *Schisostoma mansoni*, single orthologs of Wnt1, Wnt2 and Wnt5 and two paralogs of Wnt11a are present, but Wnt11b is absent. The species *Schmidtea mediterranea* possesses three paralogs of Wnt4, while other parasitic flatworms possess only one [57]. However, these results are yet to be experimentally validated.

#### Nemathelminthes

Both the canonical and noncanonical Wnt STPs exist in *C. elegans*. Its genome has five genes for Wnt ligands, four genes for Fzd receptors and one gene for Ryk/Derailed [56, 58, 59]. Unlike vertebrates or arthropods, the *C. elegans* genome has three *β*-catenin genes (WRM-1, BAR-1 and HMP-2). They have clearly demarcated functions in signaling and cell adhesion [60]. BAR-1 is a part of the canonical Wnt STP while WRM-1 is part of the noncanonical Wnt STP [59].

#### Arthropoda

Arthropods are characterized by the loss of Wnt16 [57, 61]. *Tribolium castaneum* (beetle) of Ecdysozoa super-phylum has only nine subfamilies, with no duplications [56, 58]. *Drosophila melanogaster* has just seven Wnt genes [56].

#### Echinodermata

This phylum offers insights into the earliest and the most basic of the deuterostome animals [54]. The *Strongylocentrotus purpuratus* (sea urchin) genome reveals many deuterostome-like properties. It contains genes that are absent in the more derived deuterostomes. On the contrary these particular genes are found to be present in cnidaria and/or protostomia. Hence this phylum exhibits Wnt genes present in the lower as well as higher phyla, becoming a probable link among them.

Sea urchin has eleven of the thirteen reported Wnt subfamilies along with a WntA ortholog (SpWntA) thought to be absent in deuterostomes. WntA proteins are presents in cnidarians, ecdysozoans and lophotrochozoans but have not been reported in any chordate lineage to date. From these findings, it appears that the WntA subfamily was present in the common ancestor of deuterostomes, but apparently was lost during chordate evolution [54]. Wnt2 and Wnt11 genes are absent. Loss of the Wnt-2 ortholog in Metazoa is quite an uncommon event. However, the absence of Wnt-11 is frequently encountered.

#### Chordata

Chordates are characterized by the loss of WntA. They are also known for presence of a large number of Wnt paralogs and associated downstream components [62]. Multiple Wnt antagonists, such as DKK, CER, Wnt Inhibitory Factor (WIF) and Secreted Frizzled Related Protein (SFRP) are reported in vertebrates. Evolution of complexity in Wnt signaling is probably catalyzed by the Wnt antagonists. By comparison, a few Wnt antagonists are found in basal Metazoa [43, 57]. The human genome of super-phylum Deuterostomia has 19 Wnt genes. These genes belong to Wnt1 to Wnt11, and Wnt16 [54, 63] subfamilies (12 subfamilies) with seven duplications [56, 62].

### Wnt Evolution and the Module tree

Wnt genes and the associated pathway (Wnt signaling pathway) show varied characteristics starting from placozoa (*Trichoplax adherens*) to chordata (*H. sapiens*). A complete component of Wnt signaling pathway is present in Trichoplax, irrespective of its simple body plan that presumably takes part in other functions [51–53]. The Wnt diversity continues to cnidarians which flaunt a defined body-plan indicating use of the Wnt signaling pathway. They possess 14 Wnt orthologs belonging to 12 sub-families. An additional WntA is present which do not have any human counter-part [44, 54, 55]. This diversity of Wnt genes is lost in flatworms. They possess only five sub-families of Wnts, but both the canonical and non-canonical Wnt signaling pathways are found to be functional. Wnt6-Wnt10, Wnt16 and WntA genes are lost. In addition, the Wnt4 gene is lost with rise of parasitism [57], which is probably due to easy access to the genetic machinery of the host organism.

Nematodes have only 5 Wnt ligands and flaunt more super-specialization in the form of three distinct *β*-catenin genes with distinct separate functionality [56, 58–60]. Arthropods are characterized by the loss of Wnt16 [57, 61]. The beetle *T. castaneum* (super-phylum Ecdysozoa) retains only 9 Wnt subfamilies, with no duplications [56, 58] while *D. melanogaster* (fruitfly) has just 7 Wnt genes [56]. Echinoderms retain 11 sub-families of Wnt genes with a WntA ortholog, indicating their connection with protostomes. Wnt2 and Wnt11 genes are absent. While absence of Wnt11 is quite common in other metazoans, absence of Wnt2 is an exception [54]. Chordates are characterized by their complexity in Wnt signaling, presence of multiple Wnt antagonists and loss of WntA gene. Humans have 19 Wnt genes, representing 12 subfamilies with seven duplications [56, 62].

**Fig. 6:**
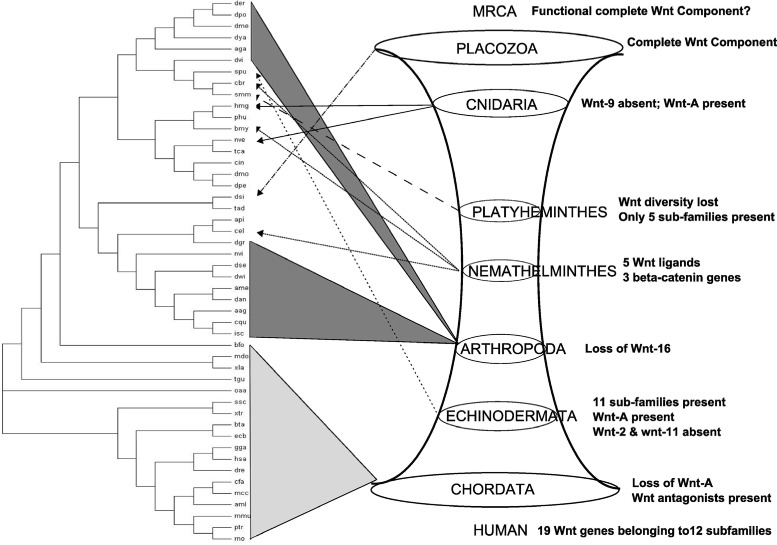
Relational aspects between module tree and Wnt signaling pathway evolution. Interestingly, Wnt genes pass through a wormhole like phase in time during evolution

These observations indicate a possible gene duplication event in MRCA ∼940 Mya (Million years ago) that continued to placozoans in a subdued manner, then lost in Platyhelminthes and Nemathelminthes. The loss is minimized in Arthropods possibly due to a gene-boom during or before divergence of Echinoderms ∼500 Mya. Echinoderms retain a mixture of old and new Wnt characteristics that flourish extensively in Chordates with loss of early Protostome characteristics [16, 64]. Presumably amidst multiple gene duplication events, the Wnt genes pass through a wormhole-like phase; wormholes being a hypothetical topological feature of space time as given in Fig. 6. Although the gene duplication events do not correlate with the origin of the principal animal groups, they can be related to evolutionary course of Wnt gene family.

When the module tree is compared with this emerging pattern of Wnt gene family evolution (Fig. 6), we have found that most of the chordates were placed in vicinity of each other, except a few. Arthropods formed two distinct clusters. Placing of the only placozoan (tad), platyheminth (smm) and echinoderm (spu) cannot be commented upon due to their singular presence in the species set. Still placing of the single platyheminth (smm) with a nematode (cbr) is justifiable from the fact that species from both the phyla tolerate Wnt diversity loss. The two cnidarians (hmg and nve) are closely placed with the echinoderm (spu) probably based on their similarity of having Wnt-A gene. The Phyla Chordata and Arthropoda are quite diverse from speciation point of view. A more compact and individual study of species of these phyla will bring more conclusive facts about Wnt signaling pathway evolution in these phyla, and may also describe the unexplained positioning of some species in the module tree.

## Conclusions

This article emphasized on development of Wnt STP over various species. Here, we have created two alternate phylogenetic trees, i.e., the pathway and module trees from four species sets of species-specific pathways (comprising 99, 48, 29 and 12 species), and compared them with two reference trees (the NCBI taxonomy tree and the 18S rRNA tree). The module tree is found to be more similar to the reference trees than the pathway tree. Hence the module tree is a better candidate to represent Wnt STP development. The increased performance of the module tree is due to consideration of local similarities, which probably we ignore in a global scenario. This concept of taking modules/sub-units/sub-sets of pathways (local information) in to construction of a phylogenetic tree rather than considering the whole pathways (global information) can be extended to other fields of phylogenetic analysis.

Moreover, the module tree has been linked with the major events that happened in course of Wnt gene family evolution. However, there are some species-arrangements in the module tree which defy the general notion of taxonomy and evolution. This may turn out to be the pressure of speciation that the phyla faced individually and originates scopes for further phylum specific research and analysis.

## Additional file

Additional file 1 It is a ‘.zip’ file containing the four figures for NCBI taxonomy, 18S rRNA, pathway and module trees for 99 species-specific Wnt STPs. Moreover, a text file titled, “the_final_trees_newick_format.txt” is provided with the newick format of these trees. (ZIP 253 kb)
