# Supplementary figures and images for: Wnt signal transduction pathways: modules, development and evolution
Source: BMC Syst Biol. 2016 Aug 1;10(Suppl 2):44. doi: 10.1186/s12918-016-0299-7 (PMC4977476; doi:10.1186/s12918-016-0299-7)

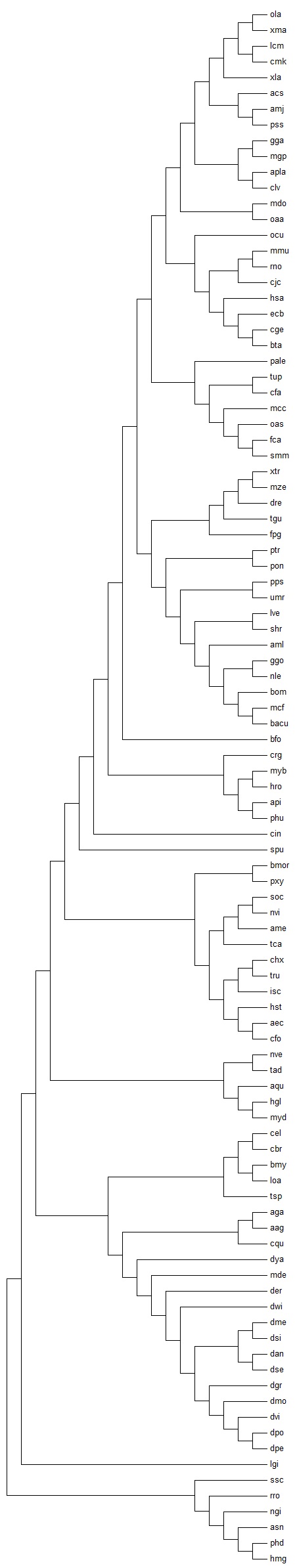

Supplement: Additional file 1 — It is a ‘.zip’ file containing the four figures for NCBI taxonomy, 18S rRNA, pathway and module trees for 99 species-specific Wnt STPs. Moreover, a text file titled, “the_final_trees_newick_format.txt” is provided with the newick format of these trees. (ZIP 253 kb) [file 12918_2016_299_MOESM1_ESM.zip › 12918_2016_299_MOESM1_ESM/18S-rRNA_99_tree.jpg]

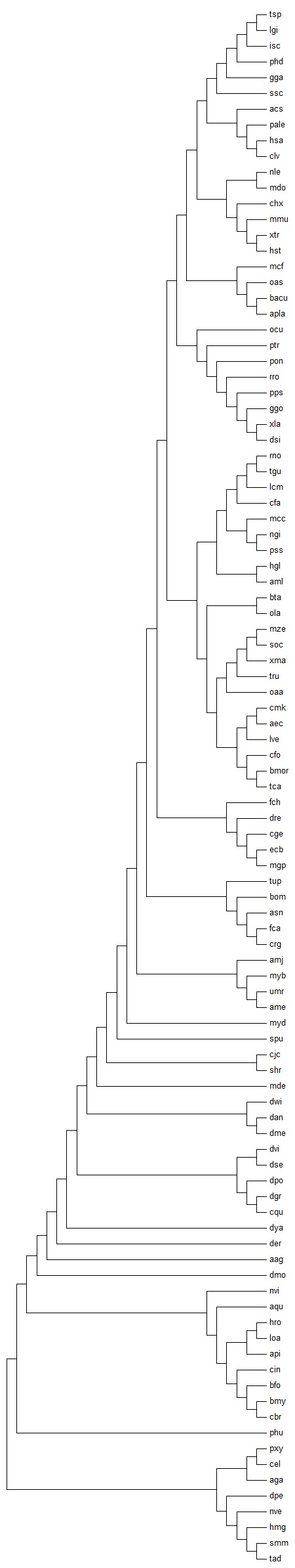

Supplement: Additional file 1 — It is a ‘.zip’ file containing the four figures for NCBI taxonomy, 18S rRNA, pathway and module trees for 99 species-specific Wnt STPs. Moreover, a text file titled, “the_final_trees_newick_format.txt” is provided with the newick format of these trees. (ZIP 253 kb) [file 12918_2016_299_MOESM1_ESM.zip › 12918_2016_299_MOESM1_ESM/graal_99_tree.jpg]

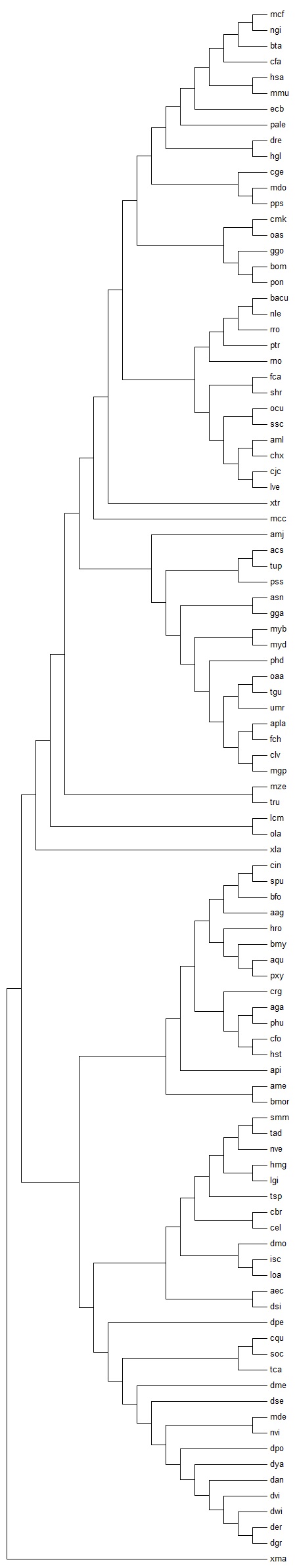

Supplement: Additional file 1 — It is a ‘.zip’ file containing the four figures for NCBI taxonomy, 18S rRNA, pathway and module trees for 99 species-specific Wnt STPs. Moreover, a text file titled, “the_final_trees_newick_format.txt” is provided with the newick format of these trees. (ZIP 253 kb) [file 12918_2016_299_MOESM1_ESM.zip › 12918_2016_299_MOESM1_ESM/module_99_tree.jpg]

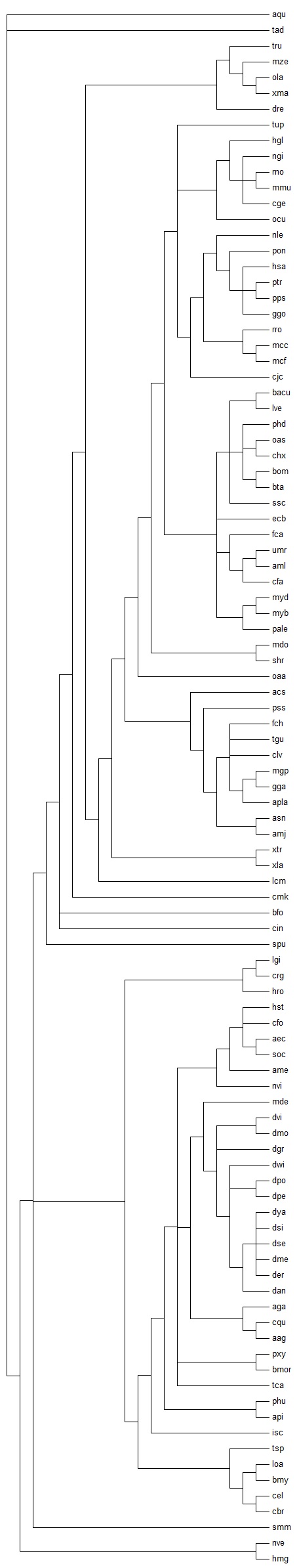

Supplement: Additional file 1 — It is a ‘.zip’ file containing the four figures for NCBI taxonomy, 18S rRNA, pathway and module trees for 99 species-specific Wnt STPs. Moreover, a text file titled, “the_final_trees_newick_format.txt” is provided with the newick format of these trees. (ZIP 253 kb) [file 12918_2016_299_MOESM1_ESM.zip › 12918_2016_299_MOESM1_ESM/ncbi_taxonomy_99_tree.jpg]
